# Integrating machine learning and statistical techniques to evaluate postural stability during hypoxia

**DOI:** 10.3389/fbioe.2026.1886164

**Published:** 2026-10-16

**Authors:** Yasaman Baradaran, Abishek Shrestha, Jonathan French, Maryam Ghahramani

**Affiliations:** 1 BioSIS (Biosensing & Intelligent Systems) Lab, Centre for Intelligent Computing and Systems, University of Canberra, Canberra, ACT, Australia; 2 Embry-Riddle Aeronautical University, Daytona, FL, United States

**Keywords:** force plate, hypoxia, machine learning, postural stability, spatial disorientation

## Abstract

This study investigates the effects of hypoxic altitude on postural stability using statistical analysis and feature-selection-based machine learning. Thirty-one healthy participants were exposed to four simulated altitudes (sea level, 3,000 m, 4,000 m, and 5,000 m) in a randomized, blinded protocol within a normobaric chamber, with arterial oxygen saturation ( ${\text{SpO}}_{2}$ ) confirming hypoxic exposure. Participants completed eight standing conditions designed to manipulate visual, vestibular, and somatosensory inputs. For each condition, 15 postural sway measures were extracted from center of pressure signals. Statistical analysis indicates that hypoxia-induced changes in postural stability are multidimensional and are most detectable under conditions that increase sensory integration demands, particularly foam based and tandem stance tasks. Moderate hypoxia increased sway variability and disrupted automatic postural control, whereas more severe hypoxia under complex task conditions elicited a constrained motor strategy. In the machine learning approach, four tree-based ensemble classifiers, including Extra Trees, Random Forest, Gradient Boosting Decision Trees, and XGBoost, were employed for altitude classification using the selected postural sway features. A feature-selection framework using Extra Trees feature importance and participant-grouped nested cross-validation was implemented. The optimized Extra Trees classifier achieved the highest accuracy of 70% using 27 selected features. The selected features highlighted the contribution of specific sway characteristics that were not fully captured by conventional univariate metrics. These findings show that moderate hypoxia, in sensory challenging environments, induces detectable multidimensional postural instability before the onset of conventionally recognized symptoms. Our results highlight the importance of task selection and multivariate analysis for detecting subtle impairments. These findings have potential applications in aerospace environments.

## 1 Introduction

Hypoxia is a well established physiological condition that arises when oxygen availability is insufficient to meet tissue demand. Exposure to high altitude reduces barometric pressure and consequently the partial pressure of inspired oxygen, resulting in hypobaric hypoxia Furian et al. (2022); Scheiwiller et al. (2022); Reiser et al. (2023); West (1998), West (2013). Reduced oxygen availability impairs cellular respiration and produces measurable physiological effects even at moderate altitudes Wagner et al. (2011). These changes degrade human performance, particularly in environments requiring precise sensorimotor control.

Hypoxia induces a range of physiological and perceptual impairments, including fatigue, dizziness, reduced coordination, and visual disturbances McMorris et al. (2017); Lieberman et al. (2005). Although loss of color vision is often cited as an early measurable symptom of hypoxia, typically occurring at altitudes around 5,000 m Smith et al., (1976), evidence suggests that functional impairments such as postural instability and SD may arise at lower altitudes Hornbein (1989). These impairments are of particular concern in operational environments such as aviation, where loss of orientation—especially in the absence of reliable visual references—can lead to catastrophic outcomes Gibb et al. (2011). Despite this, hypoxia-induced disruptions to spatial orientation remain difficult to characterize in controlled settings, where individuals are typically seated and stationary rather than actively maintaining balance Virués-Ortega et al. (2004).

Human spatial orientation depends on the integration of visual, vestibular, and somatosensory inputs within the central nervous system Weerakkody et al. (2007); Clark (2015); Zettel et al. (2005); Chen et al. (2022); Mahboobin et al. (2005); Baradaran et al. (2024). Disruption of this multisensory integration, particularly under hypoxic conditions, can result in SD and impaired postural control Zettel et al. (2005). Postural stability can be objectively quantified using measures of CoP, which reflect the combined output of neuromuscular control processes responsible for maintaining balance Raichle and Mintun (2006); Zettel et al. (2005); Mazaheri et al. (2013); Chen et al. (2021); McClenaghan et al. (1994). Alterations in CoP dynamics have been shown to reflect impairments in central sensory processing Błaszczyk (2008), making postural sway a sensitive indicator of functional instability.

Evidence from both laboratory and field studies indicates that hypoxia degrades postural stability. Experimental studies have reported increased instability during standing tasks under hypobaric and normobaric hypoxic conditions, with effects observed across both eyes-open and eyes-closed conditions Cymerman et al. (2001); Wagner, (2012); Degache et al. (2012). Field studies similarly demonstrate increased sway amplitude, velocity, and CoP path length at moderate to high altitudes Stadelmann et al. (2015); Clarke et al., (2018). Notably, symptoms associated with altitude related illness, including postural instability, have been reported at elevations as low as 1500–2000 m, with increasing prevalence above 3,000 m Fayed et al. (2006); Hackett and Roach (2001); Honigman et al. (1993); Mairer et al. (2009). These findings suggest that impairments in balance and spatial orientation may occur earlier than traditionally recognized hypoxia markers such as color vision loss.

Despite these findings, it remains unclear which specific components of postural stability and which sensory conditions are more sensitive to hypoxia. Prior studies have largely relied on a limited set of conventional sway metrics, such as CoP path length or sway area, and have typically employed univariate statistical approaches Prieto et al. (1996); Ramdani et al. (2009); Shrestha et al. (2025); Baradaran et al. (2023). These methods do not fully capture the underlying spatiotemporal and frequency domain structure of postural sway signals. The relative sensitivity of different sensory conditions—visual, vestibular, and somatosensory—to hypoxic exposure remains incompletely understood Paillard (2012); Debenham et al. (2021); Baradaran et al. (2025a). Moreover, the majority of prior studies have relied on univariate statistical comparisons or conventional regression models applied to individual sway variables Ramdani et al. (2013), which focus on gross differences at the group level Karamzadeh et al. (2016). However, multivariate machine learning classification methods are sensitive to subtle effects that may be undetectable with univariate group analysis methods. Classification methods have shown that features individually irrelevant may become relevant when used in combination in characterizing the classes Guyon and Elisseeff (2003).

Although machine learning approaches have been increasingly applied in clinical balance research, their application to hypoxia-induced postural instability remains limited. For example, machine learning techniques have been used to classify among four different classes of postural behaviors including standing, AP sway, ML sway, and unstable. This approach has demonstrated promising performance in correctly classifying unknown patterns as belonging to the right postural sway class Andò et al. (2023). Wearable IMU-based estimation of standing scores across four sensory conditions has also been investigated using multiple linear regression, support vector regression, and XGBoost, with the best lumbar-sensor XGBoost model achieving a correlation of 0.96 and an MAE of 0.23 Nassajpour et al. (2024). In one study, five machine learning algorithms, including Random Forest, XGBoost, Support Vector Machine, Logistic Regression, and Gradient Boosting, were trained to classify stability scores into three categories: High, Moderate, and Low, using demographic characteristics, clinical measures, and center-of-pressure sway. The results showed that the Gradient Boosting classifier demonstrated the best performance. Feature importance analysis identified center-of-pressure sway as one of the most influential predictors of stability classification Emam et al. (2026). Nevertheless, the application of machine learning to investigate and classify balance impairments induced by hypoxic exposure remains largely unexplored, highlighting the need for further research to develop robust predictive models for hypoxia-related postural instability.

This paper addresses these limitations and makes three primary contributions. First, it evaluates whether hypoxia-induced impairments in postural stability occur at lower altitudes than traditionally recognized symptoms such as color vision loss. Second, it identifies which sensory conditions are most sensitive to hypoxic altitude exposure through systematic manipulation of visual, vestibular, and somatosensory inputs. Third, it applies a multivariate feature-selection framework incorporating both time domain and frequency domain sway measures to provide a more comprehensive characterization of postural instability under hypoxic conditions. To achieve these objectives, 31 healthy younger participants were exposed to four simulated altitudes in a normobaric chamber, with ${\text{SpO}}_{2}$ measurements confirming hypoxic exposure. They completed eight standing conditions in which sensory inputs were selectively manipulated. For each condition, 15 postural sway measures were extracted from CoP signals. Statistical analyses and feature-selection-based machine learning were then used to identify the sway characteristics and sensory conditions most affected by hypoxia.

## 2 Materials and methods

### 2.1 Participants and experimental design

Thirty-one healthy younger adults (19–23 years) from Embry-Riddle Aeronautical University participated in this study. Participants reported no known cardiovascular, respiratory, or neurological conditions that could be exacerbated by hypoxic exposure. They provided written informed consent before participation. The sample was approximately balanced between males and females. The study was approved by the Embry-Riddle Aeronautical University Institutional Review Board and the Air Force Human Research Participants Office (#23-004). A repeated-measures design was employed in which participants completed four simulated altitude conditions: sea level, 3,000 m, 4,000 m, and 5,000 m. Altitude sessions were conducted weekly in a randomized order within a normobaric hypoxia chamber.

### 2.2 Hypoxia exposure protocol

A normobaric hypoxia chamber equipped with a computer-controlled oxygen system (Hypoxico Everest II, Europe GmbH, Germany) was used to simulate altitude exposure as shown in Figure 1. Normobaric hypoxia systems are commonly used in aviation and military training due to their physiological equivalence to hypobaric exposure and improved operational safety and efficiency Conkin (2016). Chamber oxygen concentration was continuously monitored and automatically regulated throughout each session.

**FIGURE 1:**
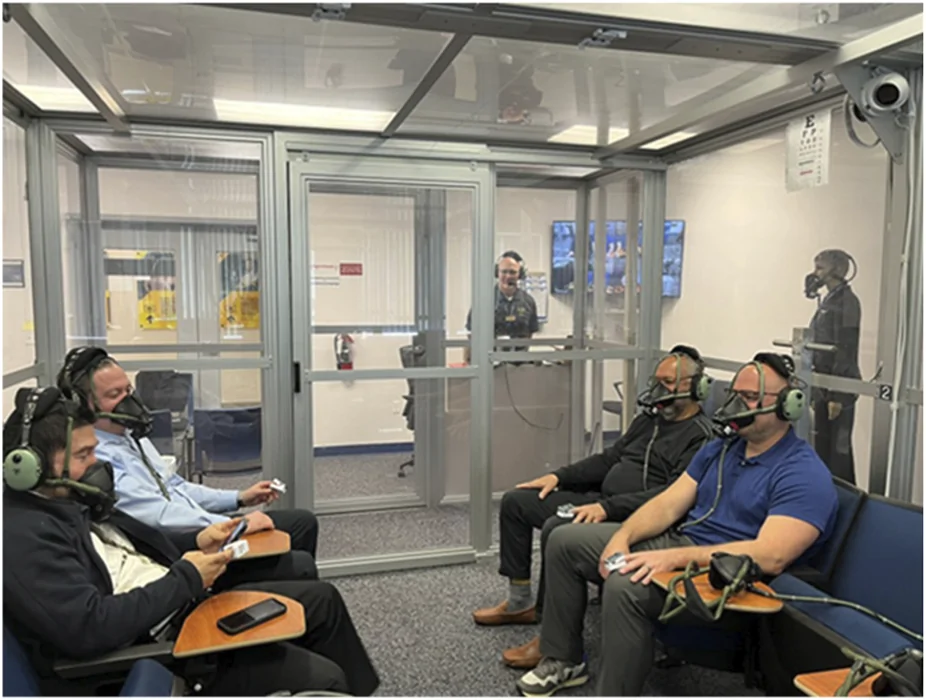
View from the south end of the HAL without the portable balance platform, showing the masks and seating arrangements. The walls of the HAL are taut, airtight Plexiglass on the frame. The 12 air scrubbers remove oxygen from the room to achieve the selected altitude. The airlock is shown separating the inside of the chamber from the outside. The console control officer is shown at the far end, outside of the HAL, at the control console.

Participants received standardized protocol and emergency briefings before exposure. During each session, participants remained seated within the chamber for a 30-min acclimation period before testing. Altitude exposure continued throughout postural testing. Peripheral arterial ${\text{SpO}}_{2}$ and heart rate were continuously monitored using a wireless fingertip pulse oximeter (Berry BM1000, Berry Electronics, China) sampled at 1 Hz to confirm hypoxic exposure and ensure participant safety throughout the experiment.

### 2.3 Postural stability assessment

Postural stability was evaluated using a modified Clinical Test of Sensory Integration and Balance (CTSIB) protocol Shumway-Cook and Horak (1986). The standing conditions were designed to selectively manipulate visual, vestibular, and somatosensory contributions to postural control. To increase vestibular challenge, the conventional visual conflict dome was replaced with continuous horizontal head movements Curthoys (2000). In addition, combined visual and vestibular perturbations were incorporated to further degrade sensory input. Participants also completed Sharpened Romberg tandem stance conditions with eyes open and closed. During tandem stance, participants stood heel-to-toe with arms folded across the chest to reduce compensatory arm movements and increase sensitivity to subtle instability Johnson et al. (2005). Each standing test lasted 30 s in accordance with clinical practice Shumway-Cook and Horak (1986). A summary of the standing conditions is provided in Table 1.

**TABLE 1:** Standing tests.

Standing test	Task
Condition 1	Normal stance
Condition 2	Standing with eyes closed
Condition 3	Standing on a foam
Condition 4	Standing on a foam with eyes closed
Condition 5	Standing on a foam while moving the head continuously from side to side
Condition 6	Standing with eyes closed on a foam while moving the head continuously from side to side
Sharpened romberg 1	Tandem heel-toe position
Sharpened romberg 2	Tandem heel-toe position with eyes closed

The potential influence of order and learning effects in these clinical balance assessments has not been fully established, particularly across repeated sessions separated by 1 week intervals. The standard clinical test order was therefore maintained, as these conditions progressively increase in difficulty from the initial to the final tasks. A within-subject design with 1 week intervals between altitude exposures was used to minimise potential order-related effects and ensure consistent exposure across conditions.

During testing, CoP data were collected using a static force platform (Sensory Kinetics, SK, Engineering Acoustics, Inc, USA) measuring anteroposterior (AP) and mediolateral (ML) sway components. The CoP data were sampled at 100 Hz, with a resolution limited to 10 bits by the microcontroller analogue-to-digital converter. The CoP measurement accuracy was approximately 2 mm within the center region of the force platform. Data acquisition and processing were performed in MATLAB (R2023b, MathWorks, USA).

### 2.4 Signal processing and feature extraction

Raw CoP signals were low pass filtered at 10 Hz using a Butterworth filter, consistent with established posturography protocols Mancini et al. (2012); Fino et al. (2019). Fifteen postural sway measures were extracted from each standing condition using both time domain and frequency domain analyses. Similar to Mancini et al. (2012) and Fino et al. (2019), extracted features included measures related to sway magnitude, variability, smoothness, and spectral characteristics. Sway measures were computed separately for AP, ML, and resultant sway directions, where resultant sway represented the Euclidean norm of AP and ML CoP displacement. A summary of extracted sway measures is presented in Table 2.

**TABLE 2:** Sway measures.

Time Domain	Frequency Domain
RMS (mm)	Mean Frequency (Hz)
AP RMS (mm)	Total Power (mm^2^)
ML RMS (mm)	ML total power (mm^2^)
Path (mm)	AP total power (mm^2^)
Distance (mm)	ML Median Frequency (Hz)
Area (mm^2^)	AP Median Frequency (Hz)
Jerk (mm/s^3^)	AP 95% power Frequency (Hz)
	ML 95% power Frequency (Hz)

### 2.5 Statistical analysis

First, a repeated-measures ANOVA with Greenhouse-Geisser correction was applied to investigate the effect of altitudes on estimated arterial ${\text{SpO}}_{2}$ and heart rate (BpM). The analysis was performed to assess whether these physiological measures significantly changed across the different altitude conditions.

Second, statistical analyses were conducted using Jamovi (Version 2.3) and Python (v3.12.2) to evaluate the effects of altitude on sway measures across standing conditions. Normality was assessed using the Shapiro-Wilk test, and homogeneity of variance was evaluated using Levene’s test. Because multiple sway measures violated normality assumptions (Shapiro-Wilk, p<.05 ), non-parametric methods were used for all analyses. Because participants completed all four altitude conditions, differences across altitudes for each standing condition and sway measure were evaluated using the Friedman test. The significance threshold was α=.05 . Kendall’s W was reported as effect size. Repeated-measures tests used the 29 participants with complete data across all four altitudes. Participants with missing altitude sessions were excluded from the Friedman and *post hoc* analyses because complete observations across all repeated conditions are required for these tests. When significant main effects were identified, *post hoc* pairwise comparisons were performed using Wilcoxon signed-rank tests. A Bonferroni correction was applied within each family of six pairwise altitude comparisons. Matched-pairs rank-biserial correlation (r) was reported as the *post hoc* effect size. Benjamini-Hochberg false discovery rate (FDR) correction was applied across all main-effect tests.

### 2.6 Machine learning classification

Machine learning classification was employed in Python (Version 3.11) using the scikit-learn library (Version 1.4.0) to classify the four altitudes using a combination of 120 sway features. The 120 candidate input features comprised 15 sway measures collected across eight standing tests. Missing values were minimal and were handled using median imputation. Because the number of candidate input features was approximately equal to the number of observations, the analysis represented a high dimensional modeling problem. Hence, to reduce overfitting, models were evaluated using incrementally larger subsets of the highest-ranked features, from the top feature to the top 30 features, rather than using all 120 predictors simultaneously.

Each participant contributed repeated observations across altitude conditions. Participant ID was therefore used as a grouping variable but was excluded from the input feature set. The data were divided into development and test sets using an 80:20 participant level split. All observations from the same participant were assigned entirely to one set, ensuring that no participant appeared in both the development and test datasets. Model development used participant-grouped 10-fold nested CV. In every fold, all observations from a participant were retained together in either the training or validation partition. Consequently, the reported validation performance represented prediction for participants who were not included in the corresponding training data.

Four tree-based ensemble classifiers—Random Forest, Gradient Boosting Decision Trees, Extra Trees and XGBoost—were evaluated. These algorithms were selected because they can model nonlinear relationships and complex interactions among sway variables, are relatively robust to multicollinearity, and generally perform well in high dimensional classification problems without requiring predictor standardization Pensa et al. (2025); Choudhury et al. (2024). Including both bagging-based methods (Random Forest and Extra Trees) and boosting-based methods (Gradient Boosting and XGBoost) also enabled comparison of different ensemble-learning strategies Wang et al. (2024); Alhams et al. (2024).

Features were ranked using importance scores derived from an Extra Trees classifier. This method was selected because its randomized tree construction can capture nonlinear and interaction effects while reducing dependence on any single tree structure. Extra Trees importance also provided a consistent ranking procedure across the four tree-based classifiers Sahin (2020); Mohammed and Hamza (2025). Feature ranking was performed separately within every outer CV training fold. The held-out participants did not contribute to feature-importance estimation, feature ranking, or feature-subset selection. Therefore, feature selection was nested within CV.

Hyperparameters were optimized within each outer training fold using ‘RandomizedSearchCV’ and participant grouped three-fold inner CV, as shown in Table 3. Fifteen classifier-specific parameter combinations were sampled, and mean inner-fold accuracy was used as the optimization criterion. The search included parameters such as the number and depth of trees, learning rate, and subsampling parameters. Randomized search was used instead of exhaustive grid search to evaluate a broad range of parameters while maintaining a feasible computational burden.

**TABLE 3:** Hyperparameter search spaces evaluated for the four classification algorithms using randomized search within participant grouped nested CV.

Classifier	Hyperparameter	Candidate values
Random forest	Number of trees (n_estimators)	100, 200
	Maximum tree depth (max_depth)	Unrestricted, 5, 10
	Minimum samples per leaf (min_samples_leaf)	1, 2, 4
	Features considered per split (max_features)	Square root, all features
Gradient boosting	Number of estimators (n_estimators)	50, 100
	Learning rate (learning_rate)	0.05, 0.10
	Maximum tree depth (max_depth)	1, 2, 3
	Minimum samples per leaf (min_samples_leaf)	1, 2, 4
Extra trees	Number of trees (n_estimators)	100, 200
	Maximum tree depth (max_depth)	Unrestricted, 5, 10
	Minimum samples per leaf (min_samples_leaf)	1, 2, 4
	Features considered per split (max_features)	Square root, all features
XGBoost	Number of estimators (n_estimators)	50, 100
	Maximum tree depth (max_depth)	2, 3, 4
	Learning rate (learning_rate)	0.03, 0.10
	Observation subsampling (subsample)	0.80, 1.00
	Feature subsampling (colsample_bytree)	0.80, 1.00

For each classifier and feature count, the mean and standard deviation of participant grouped outer-fold accuracy were calculated. The final classifier and number of features were selected using development-set CV performance, with preference given to higher mean accuracy, lower between-fold variability, and fewer predictors where results were similar.

After model selection, feature ranking and hyperparameter optimization were repeated using the complete development dataset. The held-out test set was also used to provide an additional assessment of model performance. Model and feature-count selection was based on development-set CV performance. Accuracy, precision, recall, and F1-score were used as evaluation metrics. A confusion matrix was generated to investigate the classification performance further and identify the altitudes that were most frequently misclassified.

## 3 Results

### 3.1 Statistical analysis results

Estimated ${\text{SpO}}_{2}$ decreased significantly across simulated altitude conditions, confirming effective hypoxic exposure, F(2.63,58.36)=267.2 , p<.001 (Figure 2). Post-hoc comparisons demonstrated significantly lower ${\text{SpO}}_{2}$ values at 3,000 m, 4,000 m, and 5,000 m relative to sea level (all p<.001 ). Heart rate also differed significantly across altitude conditions, F(1.903,44.41)=14.21 , p<.001 . Only the 5,000 m condition differed significantly from sea level in *post hoc* testing.

**FIGURE 2:**
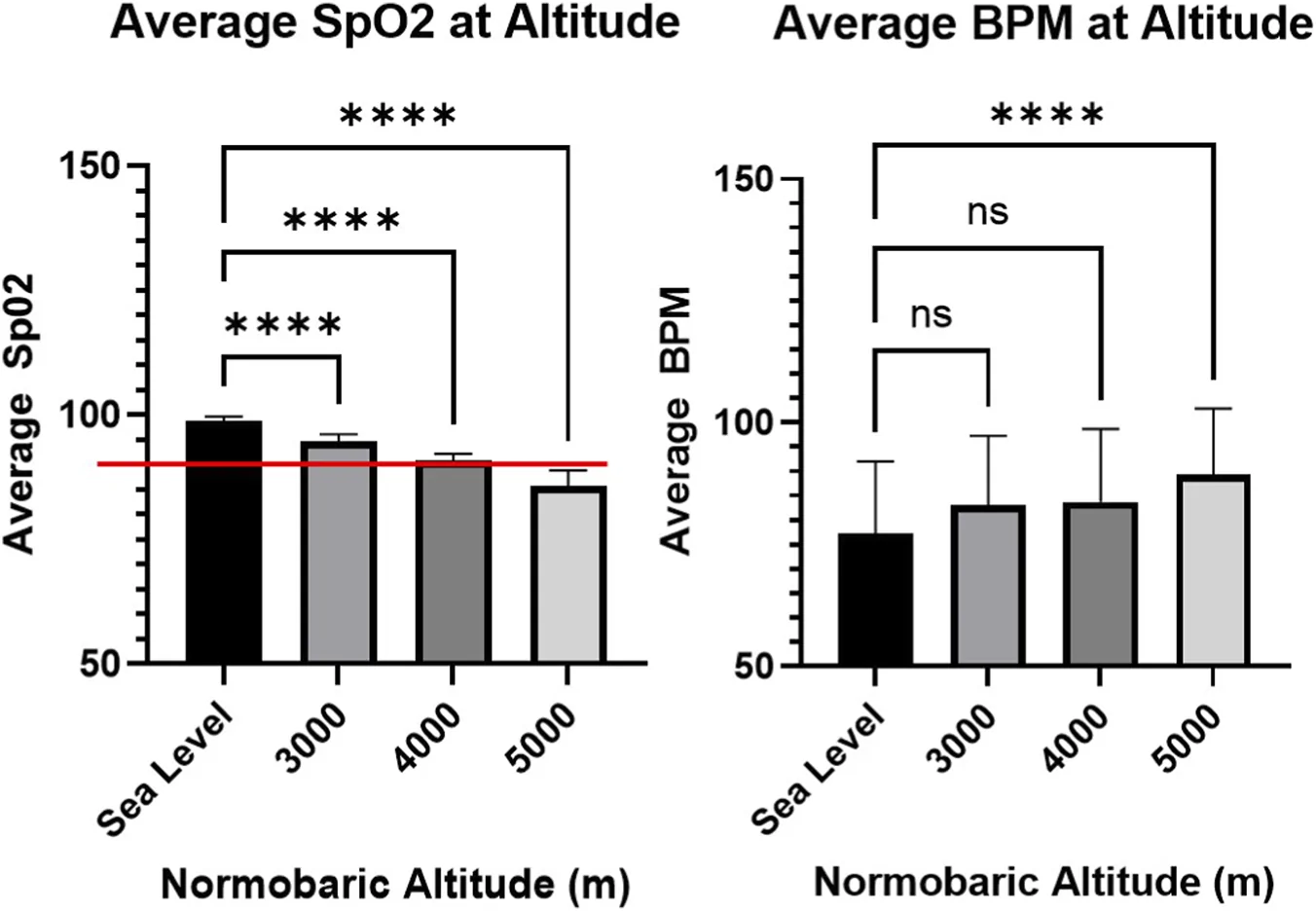
The Oximeter data recorded continuously in the hypoxia chamber showing the significant changes in estimated arterial ${\text{SpO}}_{2}$ and heart rate (BpM) at each of the altitudes tested. Data are shown as averages+/-standard deviations.

Table 4 summarizes descriptive statistics for the sway measures showing the largest (nominal, uncorrected) altitude effects. None remained significant after Benjamini–Hochberg correction for the tests performed (minimum FDR-adjusted p=.06 ). These nominal effects were concentrated in foam based and tandem standing conditions that increased sensory integration demands. The strongest nominal effect was in Condition 5 (foam surface with head movements). Sway total power was higher at 4,000 m than at sea level and at 5,000 m (Friedman $\chi^{2}\left(3\right)=17.85$ , p<.001 , Kendall’s W=.21 ; $p_{\text{FDR}}=.057$ ). Nominal effects of similar magnitude were also observed for total power in Condition 4 and Sharpened Romberg 1, for sway jerk in Condition 3, and for sway area and ML 95% power frequency in Sharpened Romberg 1. These effects were again concentrated in foam based and tandem standing conditions that increased sensory integration demands. Pairwise comparisons for these measures are presented in Table 5 and Figure 3. None of the measure × condition tests survived FDR correction, so these patterns should be interpreted as exploratory.

**TABLE 4:** Descriptive Statistics for Postural Sway Measures Exhibiting Significant Main Effects presented as Median [IQR].

Balance test	Sway measure	Altitude			
		Sea level	3000 m	4000 m	5000 m
Condition 3	Jerk ( $\times 10^{10}$ mm/ $s^{3}$ )	0.97 [2.20]	1.83 [2.75]	1.86 [5.01]	0.35 [1.28]
Condition 4	ML total Power ( $\times 10^{-5}$ ${\text{mm}}^{2}$ )	2.29 [4.19]	1.38 [1.32]	1.80 [2.18]	0.74 [1.65]
Condition 5	Total power ( $\times 10^{-5}$ ${\text{mm}}^{2}$ )	5.21 [7.65]	9.93 [8.28]	13.83 [10.75]	6.69 [5.83]
Sharpened romberg 1	Total power ( $\times 10^{-5}$ ${\text{mm}}^{2}$ )	1.36 [1.82]	1.92 [3.40]	3.17 [5.43]	2.22 [2.06]
	Area ( ${\text{mm}}^{2}$ )	0.016 [0.01]	0.09 [0.01]	0.03 [0.02]	0.02 [0.02]
	ML 95% power frequency (Hz)	0.05 [0.08]	0.02 [0.04]	0.06 [0.09]	0.05 [0.06]

**TABLE 5:** Results of the Friedman test for sway measures showing significant main effects across altitude conditions.

Standing Test	Sway Measure	$\chi^{2}\left(3\right)$	p (raw)	p (FDR)	W	Post-hoc pairs (Wilcoxon, Bonferroni)
Condition 3	Jerk	11.90	0.01	0.22	0.14	3,000 m vs. 5,000 m ( p=.007 , r=.45 )
​	​	​	​	​	​	4,000 m vs. 5,000 m ( p<.001 , r=.45 )
Condition 4	ML total power	14.90	0.002	0.11	0.17	Sea level vs. 3,000 m ( p=.001 , r=.52 )
​	​	​	​	​	​	Sea level vs. 5,000 m ( p=.001 , r=.63 )
Condition 5	Total power	17.85	<.001	0.06	0.21	Sea level vs. 4,000 m ( p<.001 , r=−.59 )
​	​	​	​	​	​	4,000 m vs. 5,000 m ( p=.001 , r=.52 )
Sharpened romberg 1	Area	11.02	0.01	0.22	0.13	Sea level vs. 4,000 m ( p=.002 , r=−.52 )
Sharpened romberg 1	ML 95% power frequency	10.33	0.02	0.22	0.12	Sea level vs. 3,000 m ( p=.002 , r=.52 )
​	​	​	​	​	​	3,000 m vs. 4,000 m ( p=.005 , r=−.46 )
​	​	​	​	​	​	3,000 m vs. 5,000 m (p=.001,r=−.46)
Sharpened romberg 1	Total power	12.19	0.01	0.22	0.14	Sea level vs. 4,000 m ( p<.001 , r=−.52 )
​	​	​	​	​	​	4,000 m vs. 5,000 m ( p=.007 , r=.52 )

The table reports the Friedman test statistic $\left(\chi^{2}\right)$ , raw and false discovery rate (FDR)-adjusted p values, Kendall’s W effect size, and significant *post hoc* pairwise comparisons from Wilcoxon signed-rank tests with Bonferroni correction. Matched-pairs rank-biserial correlation (r) is reported as the effect size for *post hoc* comparisons.

**FIGURE 3:**
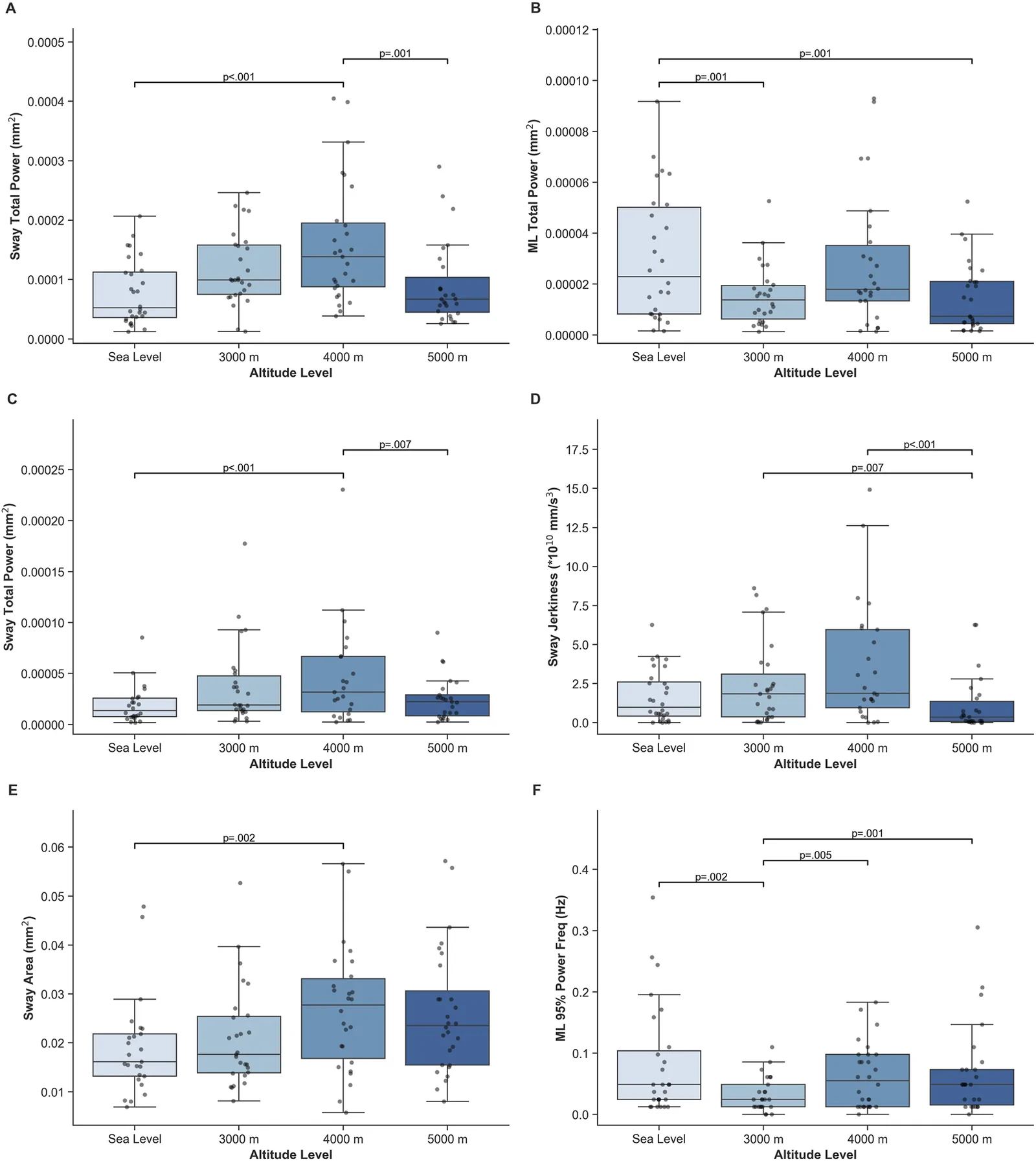
Boxplots illustrating the distribution of postural sway measures that demonstrated significant pairwise differences across different hypoxic altitudes. **(A)** Cond 5: sway total power. **(B)** Cond 4: ML total power. **(C)** Sharpened Romberg 1: sway total power. **(D)** Cond 3: sway total jerkiness. **(E)** Sharpened Romberg 1: sway area. **(F)** Sharpened Romberg 1: ML 95% power freq.

### 3.2 Classification results

Four machine learning classifiers were evaluated for altitude classification using feature-selected sway measures. Classification performance is summarized in Table 6. The highest classification performance was achieved using the Extra Trees classifier with 27 selected features, yielding an accuracy of 0.70±0.09 , precision of 0.79±0.11 , recall of 0.70±0.09 , and F1-score of 0.69±0.09 . The sway measures contributing to the highest performing model are presented in Table 7. The confusion matrix for the Extra Trees classifier is shown in Figure 4.

**TABLE 6:** Classification Results on the Development Dataset Using CV and on the independent test dataset. CV results are reported as mean ± SD across the validation folds, whereas independent test-set results are reported as single performance values.

Classifier	Evaluation	No. Features	Accuracy±SD	Precision±SD	Recall±SD	F1Score±SD
Extra trees	CV	27	0.70±0.09	0.79±0.11	0.70±0.09	0.69±0.09
	Test	​	0.67	0.73	0.66	0.69
Gradient boosting	CV	27	0.57±0.14	0.58±0.17	0.57±0.14	0.54±0.14
	​	​	0.60	0.61	0.59	0.60
Random forest	CV	18	0.47±0.07	0.50±0.11	0.47±0.07	0.45±0.08
	Test	​	0.56	0.57	0.55	0.56
XGBoost	CV	18	0.45±0.10	0.42±0.18	0.45±0.10	0.41±0.13
	Test	​	0.56	0.57	0.56	0.56

**TABLE 7:** Input features of the classification model with the best performance, based on their rankings.

Rank	Input feature	Feature importance
Rank 1	Condition 1-jerk	0.0224
Rank 2	Sharpened romberg 1-ML 95% power frequency	0.0206
Rank 3	Condition 1-AP total power	0.0196
Rank 4	Condition 5-AP total power	0.0193
Rank 5	Sharpened romberg 2-Jerkiness	0.0193
Rank 6	Sharpened romberg 1-AP 95% power frequency	0.0189
Rank 7	Sharpened romberg 1-total power	0.0185
Rank 8	Sharpened romberg 2-total power	0.0169
Rank 9	Sharpened romberg 1-ML median frequency	0.0167
Rank 10	Condition 5-Jerkiness	0.0154
Rank 11	Sharpened romberg 1-ML total power	0.0153
Rank 12	Condition 3 -jerk	0.0150
Rank 13	Condition 2 -AP total power	0.0146
Rank 14	Sharpened romberg 2-AP 95% power frequency	0.0135
Rank 15	Condition 5-ML median frequency	0.0133
Rank 16	Condition 4 -AP total power	0.0129
Rank 17	Condition 5-AP 95% power frequency	0.0126
Rank 18	Sharpened romberg 2-AP total power	0.0124
Rank 19	Condition 3 -AP total power	0.0122
Rank 20	Condition 6 -AP total power	0.0121
Rank 21	Condition 5-AP median frequency	0.0116
Rank 22	Sharpened romberg 1-AP total power	0.0115
Rank 23	Condition 1-ML total power	0.0113
Rank 24	Condition 5-Distance	0.0106
Rank 25	Condition 4 -AP median frequency	0.0104
Rank 26	Condition 1-total power	0.0104
Rank 27	Sharpened romberg 2-ML 95% power frequency	0.0100

**FIGURE 4:**
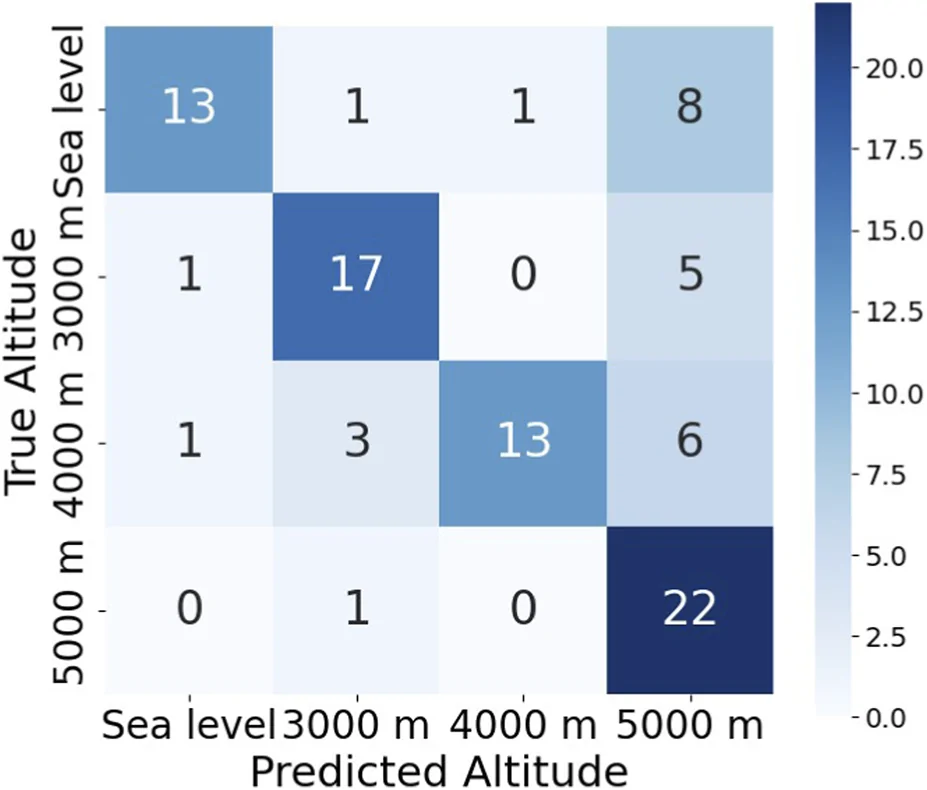
Confusion matrix based on aggregated out-of-fold predictions from participant grouped 10-fold CV for the final Extra Trees model using 27 top features.

## 4 Discussion

This study integrates conventional statistical analysis with multivariate machine learning to provide a comprehensive characterization of postural control under hypoxic conditions. By combining these complementary analytical approaches, the study enables both hypothesis driven inference and data driven pattern discovery. The results demonstrate that hypoxia-induced changes in postural stability are inherently multidimensional, with concurrent alterations across temporal, spatial, and frequency domain sway measures. These findings are directly relevant to aviation and aerospace operations, where early detection of hypoxia-related functional impairment is critical for maintaining orientation and control.

The first objective of this paper is to evaluate whether hypoxia-induced impairments in postural stability occur at lower altitudes than traditionally recognized symptoms. The results show that measurable postural instability emerges at moderate altitudes (3,000–4,000 m). This occurs potentially before the onset of more commonly recognized hypoxia-related impairments, such as color vision degradation Smith et al. (1976). These findings suggest that changes in postural control can occur during earlier stages of oxygen deprivation, even when obvious perceptual or cognitive symptoms are not yet apparent. This finding supports the use of postural stability as an early functional marker of hypoxia. The observed instability under sensory challenging standing conditions further indicates that hypoxia may compromise the integration of visual, vestibular, and somatosensory information required for maintaining balance. In operational contexts, such impairments can manifest as degraded control of heading or altitude, particularly under visually degraded conditions. Hence, they may represent a more sensitive indicator of imminent hypoxia than perceptual symptoms alone. Overall, because postural control reflects the coordinated function of multiple physiological systems, its assessment may provide a practical and objective approach for the early detection of functional degradation in aviation and aerospace environments Borden et al. (2024); Fowler et al. (1987).

The second objective of this study is to identify which sensory conditions are more sensitive to hypoxic altitude exposure through systematic manipulation of visual, vestibular, and somatosensory inputs. The results indicate that sensitivity to hypoxia is strongly task dependent. Statistical analysis shows that foam based and tandem standing conditions, which increase demands on sensory integration and postural control St George et al. (2021), were more effective in revealing instability. These findings suggest that hypoxia-related impairments become more apparent when the postural control system is challenged, and greater reliance is placed on multisensory integration mechanisms. In these conditions, elevated frequency domain measures suggest more rapid and energetically demanding corrective responses Solomonow et al. (1990). In foam based conditions, increases in time domain measures indicate reduced smoothness and stability Baradaran et al. (2025b). Together, these alterations imply that participants required more frequent and less efficient postural adjustments to maintain balance under hypoxic exposure. Machine learning analysis revealed that normal standing conditions, when combined with foam based and tandem standing conditions, can discriminate altitudes. This suggests that combining baseline and sensory challenging conditions may better capture the multidimensional effects of hypoxia on postural regulation. This further highlights the importance of machine learning approaches in identifying complex multidimensional patterns in postural behavior that may not be detectable through conventional statistical analysis alone Karamzadeh et al. (2016). On the other hand, in both statistical and machine learning analysis high demand conditions involving multiple simultaneous perturbations did not consistently produce significant effects. This suggests the adoption of constrained or stiffened postural control strategies that reduce variability and limit sensitivity to hypoxia-specific changes. Such strategies may reflect compensatory mechanisms aimed at maintaining stability under excessive sensory and physiological challenge. Hence, they hide subtle hypoxia-induced alterations in sway behavior. Overall, these findings demonstrate that sensory challenging postural tasks, particularly when combined with machine learning analysis, provide a sensitive framework for detecting subtle hypoxia-induced alterations in postural regulation and multisensory integration.

Third, in this study, the classification multivariate analysis complements the statistical findings by identifying combinations of sway measures that discriminate between altitudes more effectively than individual metrics alone. While univariate analyses identified significant changes in individual sway measures, classification results demonstrate that combinations of features provide greater discriminative power. Using the Extra Trees classifier with embedded feature selection, the highest accuracy was obtained with an optimal subset of features (Table 7), demonstrating that altitude related postural alterations are multidimensional. These findings indicate that hypoxia influences multiple aspects of postural regulation simultaneously, rather than affecting a single isolated characteristic of sway behavior. The ensemble of randomized decision trees captures non linear relationships and complex interactions among temporal, spatial, and frequency domain sway features Geurts et al. (2006). The ability of the model to integrate information across multiple domains likely contributed to its improved sensitivity compared with conventional univariate statistical approaches Karamzadeh et al. (2016). Notably, some tests—like normal standing and tandem stance with eyes closed—did not show significant effects in the statistical analysis but were revealed as sensitive to hypoxia in the multivariate classification. This sensitivity highlights the added value of feature selection embedded in machine learning approaches for detecting subtle postural deficits. This discrepancy suggests that although individual sway measures may not independently reach statistical significance, their combined patterns may still contain meaningful information related to altitude-induced functional impairment. Altogether, these findings support the use of machine learning embedded with feature selection as a complementary analytical framework for identifying complex physiological responses that may not be detectable through traditional statistical testing alone.

In this study, an additional finding is the non linear relationship between altitude and sway behavior. Both statistical and classification analyses indicate increased instability at intermediate altitudes (3,000–4,000 m), followed by reduced sway at 5,000 m. This suggests that postural responses to hypoxia may not progress in a simple linear manner with increasing altitude. Instead, they reflect distinct physiological and motor control adaptations at different stages of oxygen deprivation. This pattern is mirrored in the classification results, as shown in Figure 4. It is observed that the greatest misclassification occurs between these two altitudes, with six sea level observations predicted as 5,000 m. The overlap in classification patterns further supports the presence of complex and potentially compensatory postural responses under severe hypoxic exposure. This observation likely reflects a shift in control strategy. Moderate hypoxia appears to increase reliance on attentional resources, resulting in greater variability. However, more severe hypoxia may constrain motor output and reduce exploratory behavior. This can lead to producing superficially stable but less adaptable control patterns Nikolić et al. (2024). Such a constrained postural behavior may represent a protective or energy conserving strategy adopted under severe physiological stress. This behavior may reduce the flexibility and responsiveness required for effective balance control in operational environments. All in all, these findings highlight a non linear, stage-dependent adaptation of postural control to hypoxic exposure, reflecting a transition from increased variability under moderate hypoxia to constrained and potentially compensatory control strategies under more severe oxygen deprivation.

This study makes three key contributions: first, it shows that hypoxia-related postural instability can occur at lower altitudes than traditionally recognized, suggesting that postural instability may serve as an early functional marker of hypoxia; thereby extending current understanding of the temporal onset of hypoxia-induced motor impairments. Second, it identifies specific standing conditions and postural sway measures that are sensitive to altitude-induced hypoxia, highlighting which aspects of postural stability are most affected; and providing a more detailed characterization of task dependent and measure-specific sensitivity to oxygen deprivation. And third, by integrating both statistical and multivariate machine learning approaches, the study moves beyond traditional univariate methods, providing a more comprehensive understanding of how multiple sway characteristics change simultaneously under hypoxic conditions. This multimethod framework enables the detection of both isolated and interacting effects across postural control features, improving sensitivity to subtle physiological changes. The findings of this study are particularly relevant for aerospace environments, where crew may experience hypoxia during unpressurized flight, cabin depressurization, or periods of high workload, and could inform early detection strategies and the use of supplemental oxygen to maintain aircraft orientation and prevent loss of horizon. However, further validation in operational populations is required before these findings can inform aviation procedures or supplemental oxygen requirements. Overall, these results support the potential integration of objective postural monitoring into operational safety systems to enhance early warning capabilities in hypoxia-prone environments.

Several limitations should be considered. First, the sample size was modest and limited to healthy younger individuals, which may restrict generalizability to broader populations with greater diversity in sex, gender, age, and occupational characteristics. Second, the study was conducted under normobaric hypoxic exposure, and differences between normobaric and hypobaric hypoxia may influence physiological responses and should be considered when extending these findings to real-world aviation environments. The cross-sectional design does not capture adaptation or acclimatization effects. Third, model interpretability analyses, such as SHAP values or other model agnostic interpretability approaches, were not performed and should be incorporated in future work to provide deeper insight into the contribution of individual features to model predictions. Fourth, the current study focused on conventional scalar sway features, which may not fully capture the temporal organization and nonlinear characteristics of postural control. Fluctuation and recurrence quantification analysis can be considered in future work to identify subtle changes in postural control dynamics under hypoxic conditions. Future studies should further investigate whether objective postural stability measures can provide evidence to inform potential adjustments to aviation oxygen requirements, including potential reductions in these thresholds. They can include larger and more diverse cohorts, longitudinal designs, external validation, and validation in operational environments. Integration with neurophysiological measures, such as brain electrical activity or functional imaging, may further elucidate the mechanisms underlying hypoxia-induced impairments and support the development of more comprehensive approaches for assessing hypoxia-related functional degradation.

## 5 Conclusion

This study demonstrates that hypoxia-induced alterations in postural stability are multidimensional, affecting temporal, spatial, and frequency domain sway characteristics. Instability emerges at moderate altitudes (3,000–4,000 m), indicating that postural control may serve as an early functional marker of hypoxia. Foam based and tandem standing tasks were most sensitive to these effects, reflecting increased demands on sensory integration and central processing, while normal standing conditions remained relatively unaffected. Multivariate machine learning approaches identified subtle changes not captured by conventional statistical methods, emphasizing their value for early detection of functional impairment. The results further suggest that moderate hypoxia increases variability through greater reliance on attentional resources, whereas severe hypoxia constrains motor output, producing superficially stable but less adaptable control strategies. These findings have direct implications for aviation, where hypoxia is a known contributor to SD and in-flight incidents, particularly under visually degraded conditions. The results indicate that SD may be affecting pilots at altitudes thousands of meters below where aviation regulatory agencies require supplemental oxygen. Objective postural stability measures may provide a sensitive tool for early detection of hypoxia-related impairment, potentially preceding traditional perceptual symptoms such as color vision loss. More broadly, this work establishes a framework for using hypoxia as a model to investigate neural mechanisms of impaired sensorimotor control in operational environments. Importantly, integrating machine learning with conventional analytical approaches demonstrates the potential of data driven methods to detect subtle and complex physiological changes that may not be apparent through traditional statistical analyses alone. These findings highlight the growing importance of machine learning in aerospace and biomedical applications, where early and reliable identification of functional impairment is essential for improving operational safety, decision-making, and human performance monitoring.

## Data Availability

The raw data supporting the conclusions of this article will be made available by the authors, without undue reservation.
